# The Dual Role of HLA-G in Cancer

**DOI:** 10.1155/2014/359748

**Published:** 2014-03-31

**Authors:** Nathalie Rouas-Freiss, Philippe Moreau, Joel LeMaoult, Edgardo D. Carosella

**Affiliations:** ^1^CEA, Institut des Maladies Emergentes et des Therapies Innovantes (IMETI), Service de Recherche en Hemato-Immunologie (SRHI), Hopital Saint-Louis, 75010 Paris, France; ^2^Université Paris Diderot, Sorbonne Paris Cité, IUH, Hopital Saint-Louis, UMR_E5, 75010 Paris, France

## Abstract

We here review the current data on the role of HLA-G in cancer based on recent findings of an unexpected antitumor activity of HLA-G in hematological malignancies. For the past decade, HLA-G has been described as a tumor-escape mechanism favoring cancer progression, and blocking strategies have been proposed to counteract it. Aside from these numerous studies on solid tumors, recent data showed that HLA-G inhibits the proliferation of malignant B cells due to the interaction between HLA-G and its receptor ILT2, which mediates negative signaling on B cell proliferation. These results led to the conjecture that, according to the malignant cell type, HLA-G should be blocked or conversely induced to counteract tumor progression. In this context, we will here present (i) the dual role of HLA-G in solid and liquid tumors with special emphasis on (ii) the HLA-G active structures and their related ILT2 and ILT4 receptors and (iii) the current knowledge on regulatory mechanisms of HLA-G expression in tumors.

## 1. Discrepancy in the Role of HLA-G in Solid and Liquid Tumors

**Figure 1:**
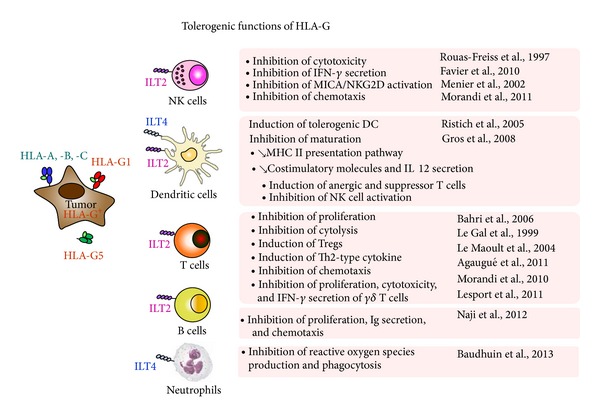
Tolerogenic functions of HLA-G.

Although various immune effector cells are recruited to the tumor site, their antitumor functions are downregulated, largely in response to tumor-derived signals [1]. In this context, expression of the tolerogenic HLA-G molecule represents a mechanism that may favor tumor survival through interaction with inhibitory receptors. We will here focus on the ILT2 and ILT4 inhibitory receptors that are present on NK, T, B, dendritic cells, and neutrophils in which they mediate negative signaling that counteracts immune activation (Figure 1). The result is tumor escape from the host immune system [2]. Thus, understanding such mechanism is an important challenge in order to develop optimal immunotherapeutic strategies.

HLA-G has been shown to be expressed in many types of primary solid tumors and metastases and in malignant effusions [3]. HLA-G can be found on tumor cells as well as on tumor-infiltrating cells [4]. The clinical relevance of HLA-G in cancer is supported by the following observations: (i) HLA-G expression is associated with malignant transformation and is never observed in healthy surrounding tissues [5]; (ii) HLA-G is found to be expressed in solid tumors of high histological grades and advanced clinical stages [6, 7]; and (iii) the use of HLA-G as a prognostic marker has been proposed since HLA-G expression in biopsies and/or high levels of soluble HLA-G (sHLA-G) in plasma from patients have been significantly correlated with poor prognosis [6–11]. All these data highlight a role for HLA-G in the immune surveillance of solid tumors and the progression of the disease.

Regarding the relationships between tumor and immune system, the concept of cancer immunoediting has been described as an important host protection process that includes three essential phases: elimination, equilibrium, and escape [12]. HLA-G can interfere with each of these phases. Indeed, (i) HLA-G can downregulate the elimination phase by inhibiting the proliferation of T and B cells, the cytotoxic activity of NK cells and CTL, the phagocytic activity of neutrophils, and the function of DC, via ILT2 and ILT4 signaling [3, 13–21] (Figure 1). In this phase, HLA-G expression would enable a proportion of tumor cells to evade the host immune response. (ii) Proinflammatory cytokines such as IFN*γ* which are secreted in high amounts may upregulate HLA-G expression [22]. HLA-G could also affect the equilibrium phase by controlling the expression of HLA class II molecules by DC [23]. (iii) In the evasion phase, tumor cells have lost molecules important for the immune recognition and tend to express only HLA-G on the cell surface, rendering them less susceptible to effector cells. The resulting rapidly growing tumors create a hypoxic microenvironment which promotes angiogenesis, invasion and metastases, but also induces HLA-G expression on tumor cells. Additionally, the immunosuppressive cytokine IL-10 which is produced in high quantities during this phase upregulates HLA-G expression [24]. Both IL-10 and HLA-G may be produced by tumor cells but also by tumor-infiltrating leucocytes [25]. Finally, HLA-G has been shown to induce regulatory T cells reinforcing tolerogenic environment [26–29]. All these mechanisms may profoundly alter antitumoral immune responses leading to tumor expansion and spread through blockage of both innate and adaptive immunity and by inducing tolerance to the tumor.

Recently, the development of animal models established the proof of concept that an HLA-G^+^ tumor cell can develop and tolerize the host antitumor immune response* in vivo *[30, 31]. Although there is no murine homologue of HLA-G,* in vivo* studies were made possible by the fact that human HLA-G can bind and mediate a signal via the murine receptor Paired immunoglobulin-like receptor (PIR)-B, the homologue of human ILTs [23, 32]. Results showed that human or murine tumor cells expressing HLA-G can grow in an immunocompetent host and that blocking HLA-G function by a specific antibody inhibits the development of the tumor. Characterization of the mechanisms by which HLA-G operates* in vivo* shows that both innate and adaptive immunity are affected [30, 31]. Hence, tumor proliferation is accompanied by an expansion of CD11b+ Gr1+ myeloid-derived suppressor cells, loss of peripheral T cells, and a cytokine balance in favor of a Th2 profile versus Th1/Th17. Therefore, blocking HLA-G may constitute a novel innovative antitumoral approach [2, 33].

Whereas HLA-G expression and its clinical significance in solid tumors have been extensively investigated showing that HLA-G expression in such tumor cells has unfavorable outcome or prognosis [34]; only a few data are available for the HLA-G expression and its clinical significance in liquid malignancies. In this regard, enhanced sHLA-G (i.e., HLA-G5 and shed HLA-G1) plasma levels have been described in B cell malignancies, such as multiple myeloma, non-Hodgkin B-lymphoma, and B-CLL [35, 36]. However, no clear correlation was established between HLA-G and unfavorable clinical outcome in hematological malignancies. Several factors may account for these controversial results such as clinical status, chemotherapy, and methodological differences between the studies.

Of note, discrepancy between solid and liquid tumors may be due to the nature of malignant cells: in hematological malignancies, tumor cells are immune cells able to express HLA-G receptors and HLA-G may have an unexpected role through inhibition of neoplastic cell proliferation. In this regard, we recently demonstrated that proliferation of hematological tumors expressing HLA-G receptor (ILT2) may be inhibited by HLA-G [37]. Indeed, HLA-G inhibits the proliferation of human B cell lymphomas, myelomas, and B cell leukemia expressing at their surface the receptor ILT2. Blocking HLA-G or its receptor ILT2 by specific antibody or siRNA could restore B cell proliferation demonstrating the role played by the interaction between ILT2 and HLA-G in the antiproliferative activity observed. HLA-G was found to reduce B cell proliferation by inducing a G0/G1 cell cycle arrest. Signaling events leading to HLA-G inhibitory effects on malignant B cells were mediated through increased PKC*α*/*β*II, PKC*δ*, and PKC*μ* phosphorylation and decreased phosphorylation of AKT, mTOR, GSK-3*β*, c-Raf, and Foxo proteins. All these effects converge to activate inhibitors or to inhibit activators of cell survival, growth, and proliferation. Indeed, PKC*δ* and PKC*μ* phosphorylated active forms have been described to negatively regulate B cells [38]. In addition, the mTOR pathway is a critical part of the cellular circuitry which is often constitutively activated in tumor cells. Our data provide evidence that HLA-G may inhibit tumor cell proliferation through mTOR signaling blockade. These results were consolidated by studies using bone marrow specimens from myeloma patients in whom HLA-G limits CD138^−^ stem cell differentiation into CD138^+^ myelomatous tumor cells. Of note, we previously described the production of soluble HLA-G proteins by bone marrow-derived mesenchymal stem cells [29] as well as by osteoblasts [39]. Analysis of cross-talk between tumor B cells, HLA-G^+^ mesenchymal stem cells, and osteoblasts in bone marrow may provide insights in understanding the mechanisms of tumor suppression* in vivo* in this biological compartment [40].

These data indicate a particularly innovative use of HLA-G in cancer since we propose a radically opposite action of HLA-G if the tumor cell is an immune system cell, the function of which could be affected by HLA-G due to the presence of surface inhibitory receptors for HLA-G. This does not involve blocking HLA-G to favor antitumoral response against solid tumor but, conversely, using the antiproliferative properties of HLA-G to limit tumor progression for hematological malignancies. Although, we are describing the role of HLA-G on B-cell malignancies, we can expect a similar effect on malignant blood diseases involving T cells, NK cells, and monocytes since these fulfil the same criteria as B cells (i.e., cell-surface expression of HLA-G receptors with inhibitory functions).

In agreement with this HLA-G antitumor activity in B cell malignancies, we previously showed that soluble HLA-G inhibits the growth of erythropoietin (EPO)-independent colonies in patients suffering from* polycythaemia vera *[41, 42]. This myeloproliferative syndrome is characterized by erythroid cells displaying deregulated proliferation due to a mutation (V617F) in the signaling protein JAK2, inducing autoactivation of this kinase by autophosphorylation [43]. As JAK2 is directly related to the EPO receptor, this increase in activity leads to constitutive signaling of the EPO receptor and leads to the formation of EPO-independent erythroid colonies, one of the biological criteria of this disease. The patient presents proliferation of erythroid cells in the bone marrow which gives rise to hyperproduction of red blood cells, leading to polycythaemia in the peripheral blood. This disease has serious consequences since it may progress towards myelofibrosis with splenomegaly, or indeed leukaemia. Hence, the ability to thwart the effects of JAK2 V617F mutation could represent a major therapeutic benefit. Our research shows that the soluble HLA-G protein acts on protein kinase JAK2 by inducing its dephosphorylation. Notably, none of the known HLA-G receptors was found on erythroid cells leading to the hypothesis that HLA-G may act on this lineage through an unidentified receptor. Hence, soluble HLA-G may be perceived as a new negative regulator for the EPO receptor signaling and may therefore represent a potential therapeutic agent for the treatment of patients carrying the mutation JAK2 V617F found not only in* polycythaemia vera* but also in the other two main myeloproliferative syndromes, essential thrombocythemia and idiopathic myelofibrosis.

The HLA-G primary transcript is alternatively spliced leading to seven splice variants encoding 4 membrane-bound (HLA-G1 to -G4) and 3 soluble (HLA-G5 to -G7) protein isoforms. Although both membrane-bound and soluble HLA-G forms exert similar immunosuppressive functions through binding to ILT receptors, the soluble forms constitute the most relevant and adapted tools for therapeutic use. Nevertheless, the main obstacle to the implementation of soluble HLA-G as therapeutic is the absence of a form that is simpler than the complex trimolecular heavy chain/B2M/peptide with which the majority of data on HLA-G were obtained. Current knowledge on the multiple HLA-G active structures and their potential use in therapy is described in the following section.

## 2. The Multiple HLA-G Active Structures and Their Corresponding Receptors

What is the actual structure of tolerogenic HLA-G is an important question. With very few exceptions, the only HLA-G structures that are currently investigated,* in vitro* and* in vivo*, are B2M-associated isoforms. Consequently, only the HLA-G/ILT2 interaction is taken into consideration since ILT4 binds mostly B2M-free isoforms. Because it was clearly demonstrated that HLA-G dimers carry most if not all of HLA-G immune-inhibitory functions [44–46], it is fair to state that currently, “tolerogenic HLA-G” is “dimeric B2M-associated HLA-G that acts through ILT2.”

This conception, which originates from the translation of* in vitro* experiments to the* in vivo* setting, is accentuated by the lack of analytic tools (especially antibodies) capable of specifically detecting anything more precise than “B2M-associated HLA-G,” “unfolded HLA-G,” or “secreted HLA-G,” and might be incomplete at best. First of all, B2M-associated HLA-G and its interaction with ILT2 might not be the most important, or the most functional HLA-G structure* in vivo.* Indeed, cytotrophoblast cells express HLA-G heavy chains that are not associated with B2M for the lack of expression of this protein [47, 48]. Therefore, if the assumption holds true that HLA-G exerts its primary function at the fetal-maternal interface and that HLA-G functions in an identical fashion in other contexts in the adult, B2M-free HLA-G should bear high significance in the periphery as well. In this regards,* in vitro* studies demonstrated that B2M-associated HLA-G and B2M-free HLA-G molecules were identically capable of inhibiting the alloproliferation of T cells in mixed lymphocyte reactions [27]. Knowing that B2M-free HLA-G binds ILT4 but not ILT2 [45, 49], it may be that ILT4 and not ILT2 is the receptor most relevant to HLA-G* in vivo* function. This notion is strengthened by recent data showing that ILT4 is the receptor for dimers of the alpha1-alpha3 structure of HLA-G (HLA-G2 and HLA-G6 isoforms) that is tolerogenic* in vivo* in murine skin transplantation models [50].

Atop of variations in its basic backbone and association with B2M, HLA-G can undergo posttranslational modifications. (i) It is long known that HLA-G can be glycosylated, especially when produced by trophoblast cells [51]. More recently, (ii) it was demonstrated that HLA-G can also be nitrated [52]. As nitration is produced at the site of nitric oxide production where the immune system is activated, HLA-G produced at sites of inflammation could be distinguished from HLA-G produced elsewhere. (iii) Due to ubiquitination, HLA-G can form high molecular weight complexes through disulfide bridges. These high molecular weight complexes are present in exudates from patients with inflammatory diseases or cancer and may also bear pathology-specific significance [53].

The notion that the function of HLA-G may depend on additional molecules is barely considered. Yet, given the diversity of HLA-G functions, it is very likely that all of them are not solely due to the interaction of HLA-G with its inhibitory receptors. It is entirely possible that the function of HLA-G is boosted by a synergy with other molecules, or reduced by others. In this regard, molecules that are functionally linked to HLA-G are already known. For instance, IL-10 is an inducer of HLA-G expression and is induced by HLA-G [24, 29, 54]. Similarly, indoleamine 2,3 dioxygenase (IDO) expression and/or function was shown to modulate and to be modulated by HLA-G [55, 56]. Amongst molecules that may deeply affect the function of soluble HLA-G are soluble HLA-G inhibitory receptors. Indeed, binding of soluble ILT2 or ILT4 to HLA-G is likely to block HLA-G function. Thus, parameters of the microenvironment might be crucial to HLA-G function. In addition, recent data confirm the original observation [57] that HLA-G is present in exosomes, in the culture supernatants of bladder cancer cells, and within exudates [53] and plasma (see Rebmann in [58]) of cancer patients. Exosomes are immune-active microparticles* per se*, either because of the external proteins they display or because of their internal immunomodulatory content or both. The actual function of HLA-G-loaded exosomes is yet to be elucidated, but it is likely that,depending on the exosome composition, HLA-G function might vary in terms of efficiency, and/or nature. Thus, exosome-bound and not-exosome-bound HLA-G may bear different pathological significance.

For lack of tools to specifically detect any of the HLA-G structures mentioned above, all get pooled into the “B2M-associated,” “unfolded,” or “secreted” categories, and, thus, their specific characteristics are not considered.

Analysis of the receptors that are responsible for HLA-G function in vivo represents also a critical point. This question may seem trivial since it is commonly admitted that HLA-G has two main inhibitory receptors: ILT2 and ILT4, that these are differentially expressed by all immune cells, and that they recognize different structural features of HLA-G (for review, see [59]). Yet, the situation might not be so simple as shown by a very simple fact: only 0–5% of CD4^+^ T cells, 10–15% of CD8^+^ T cells, and 20–25% of NK cells from PBMC express ILT2. How then, in mixed lymphocyte reactions and allocytotoxicity experiments, can HLA-G reliably inhibit PBMC T cells and NK cells directly through ILT2, ILT2^+^ cells and ILT2^−^ cells alike? One possibility is that HLA-G does not act directly on the T cell or NK populations but on APCs (B cells of myeloid) which all express HLA-G receptors. Another possibility is that HLA-G does act directly on the T and NK cell effectors, but not through the ILT2 receptors that they express. We investigated this latter hypothesis.

In a first set of studies, we evaluated the capability of HLA-G to act through the mechanism of trogocytosis, that is, the transfer of membrane fragments and the proteins they contain from one cell to another. This work revealed that HLA-G can be transferred from cells that express it to cells that do not and still exert its function [60, 61]. We also demonstrated that not only HLA-G but also ILT2 could function through trogocytosis: ILT2-negative T cells acquired ILT2 from monocytes, transiently becoming ILT2^+^ T cells that could use this borrowed receptor to respond to HLA-G direct stimulation [62]. Thus, trogocytosis of HLA-G receptors can explain the general inhibitory effect of HLA-G through known receptors on a cell population that in majority does not produce them endogenously, as long as some HLA-G receptor-expressing cells are present.

In a second set of studies, we investigated whether HLA-G could act through yet unknown receptors and we demonstrated, in systems where trogocytosis of HLA-G receptors was not possible, that cells which did not express HLA-G receptors could still respond to HLA-G stimulation. For instance, we demonstrated that HLA-G induced the upregulation of inhibitory receptor transcription in several immune cell lines including the KG1a promyeloblast line, which did not transcribe the known HLA-G receptors [63]. In another study, we investigated the capability of synthetic HLA-G proteins to inhibit the cellular multiplication of hematological tumor cell lines. This had previously been reported for membrane-bound HLA-G1 and soluble HLA-G5 and was shown to occur through HLA-G/ILT2 engagement [37, 61]. We found that HLA-G synthetic proteins which were not capable of binding ILT2 could nevertheless inhibit the cellular multiplication of hematological cell lines. Furthermore, HLA-G synthetic proteins were shown to inhibit cell lines that did not even express known HLA-G receptors [42, 64]. The identification of such a novel HLA-G receptor and of its associated functions, the possible functional associations, or overlaps with the already known ones is crucial to HLA-G biology and to the clinical applications of this molecule, as well as to the determination of its cellular expression pattern.

In addition to the need of better defining* in vivo* active HLA-G structures and their interactions with other molecules from the microenvironment, the last part of this review is dedicated to the regulation of* HLA-G* expression, an additional critical point to elucidate in the context of tumors.

## 3. The Control of HLA-G Expression

In healthy tissues, HLA-G molecules are absent with the exception of placenta [65, 66], thymus [67], pancreas [68], cornea [69], proximal nail matrix [70], and organs sustaining erythropoiesis [41]. Nevertheless, ectopic expression of HLA-G is commonly observed in pathological situations such as cancer, not rejected allografts, virus infections, autoimmune diseases, and inflammation [3]. In both normal and pathological contexts, the presence or absence of HLA-G is generally independent of classical HLA class I molecules and involves transcriptional, posttranscriptional, and posttranslational mechanisms.

Among key regulatory processes involved in HLA-G expression, DNA methylation (CpG) and hypoacetylation of H3 and H4 histones at the HLA-G locus have been associated with the absence of HLA-G expression commonly observed in cultured tumor cells expressing or not classical HLA molecules. The HLA-G gene repression can be reversed by demethylating agents used in some cancer therapies such as 5-aza-2′-deoxycytidine [71, 72] and inhibitors of histone deacetylases [72] and may directly induce HLA-G cell surface expression.

HLA-G transcriptional activity is also controlled by a unique machinery since almost all known regulatory sequences for classical HLA class I gene are disrupted for HLA-G gene, including some that are shared with HLA class II genes [73]. Alternative regulatory elements have been identified such as a putative locus control region (LCR) contained in a 250 bp fragment at 1.2 kb from the ATG translation initiation site [74]. The LCR was shown to be critical for spatiotemporal HLA-G transcription in transgenic mouse placenta [75]. Among factors demonstrated to target this region, CREB1 factor was described by the group of Peter van den Elsen to bind two cAMP response elements (CRE) at positions −1381 and −1371 (3′ ends) from the ATG [76]. CREB-1 was also demonstrated to bind two additional CREs scattered through the distal promoter region at positions −935 and −771 [76]. In addition, we reported the location of a functional binding site for IRF-1 (−744) in response to IFN-beta [22] and binding sites for heat shock factor 1 (HSF1) (−459/−453) in response to heat-shock or arsenate treatments [77]. Besides these regulatory elements, a binding site for the progesterone receptor (PR) was located at position −38 [78]. Recently, HLA-G repressors have been identified by our group and others, including Ras responsive element binding protein 1 (RREB-1) which binds three Ras response elements (RRE) dispersed along the HLA-G proximal and distal gene promoter (−1357, −143, and −54) [79], GLI-3 factor (−1108) [39], and a LINE-1 element in which 3′ end is located at −4 kb [80]. Interestingly, a hairpin loop can form in the LINE-1 element leading to HLA-G silencing [80].

HLA-G expression is also controlled at the posttranscriptional levels by mechanisms targeting the 3′ untranslated region (3′ UTR) that affect mRNA stability [78, 81] and by specific microRNAs (miR-133a, miR-148a, miR-148b, and miR-152) [82, 83]. At the posttranslational level, HLA-G expression is regulated by the antigen presenting machinery (APM) [84, 85] and a specific quality-control process mediated by the truncated HLA-G tail that control high affinity peptide loading and increases HLA-G cell-surface expression [86].

Environmental factors commonly found in placenta or tumors are certainly crucial in the induction, upregulation, and maintaining of HLA-G expression since HLA-G^+^ cells may lose HLA-G expression during long term cultures in standard conditions [87]. These factors include cytokines (IL-10 [24], IFN-*γ* [88], IFN-*γ*+GM-CSF, IFN-*γ*+IL-12 [89, 90], IFN-*β* [90], and LIF [91]), hormones (dexamethasone, hydrocortisone [92], and progesterone [92]), galectin-1 [93], IDO [56], and stress conditions (heat shock [90] and hypoxia [94, 95]). These regulatory molecules exert a control on the amounts of HLA-G transcripts and/or HLA-G protein and for some may have the opposite effect (downregulation) on classical HLA-class I expression (IL-10 [24], glucocorticoid hormones [92]).* In vitro* experiments reveal that most of them require* HLA-G* basal transcriptional activity to have an effect. Among environmental factors able to reverse* HLA-G* repression, hypoxia, which is a common stress condition (cancer, transplantation, and pregnancy), is considered as a critical candidate [94, 95].

Otherwise, several lines of evidence are reported for balancing selection acting on the 5′ and 3′ untranslated regions of the HLA-G gene (5′ UTR and 3′ UTR) [96, 97]. Considering 46 HLA-G alleles (50 alleles have been recognized to date), six different HLA-G lineages are identified showing variations mainly in the regulatory regions [97]. This indicates that these lineages are probably related to different expression profiles depending on microenvironmental factors and on physiological or pathological conditions.

Interestingly, at least 33 single nucleotide polymorphisms (SNP) are described in the 5′ UTR, defining at least 11 haplotypes [96–100]. They can modify potentially methylated CpG sites and are within or close to known or putative regulatory elements and thus might influence transcriptional activity [73, 100–102]. For instance, it should be noted that (i) a CpG site at positions −965/−964 may be affected by the presence of an Adenine at position −964 (0.5 frequency); (ii) the −56 C-T polymorphism coincides with the RREB-1 binding site located in the proximal HLA-G promoter region and is close to the PR element located at position −38 (iii) −762 C-T and −725 C-T-G polymorphisms surround the ISRE at position −744. Nonetheless, very few studies have investigated the impact of HLA-G promoter polymorphisms on the level of HLA-G expression. In particular, using luciferase reporter assays performed in JEG-3 choriocarcinoma cell line showed that a Guanine instead of a Cytosine or Thymine at nucleotide position –725 in the promoter region of HLA-G results in increased transcription rates [101].

Several polymorphic sites are also identified in the 3′ UTR, a 14 bp insertion/deletion (Indel) [103] and 7 SNPS (+3003T-C, +3010C-G, +3027A-C, +3035C-T, +3142G-C, +3187A-G, and +3196 C-G), defining at least 7 haplotypes with frequency >0.05 [104]. The presence/absence of the 14 bp sequence has been associated with the magnitude of HLA-G production in normal and pathological situations [100]. In particular, alleles presenting the 14 bp sequence are associated with reduced HLA-G mRNA and lower levels of sHLA-G in the plasma from healthy subjects [105–108]. Moreover, a minor fraction of HLA-G transcripts generated by the 14 bp alleles can be further processed by the removal of a 92 b fragment containing the 14 b sequence. These transcripts are more stable than the complete form and might be the consequence of the presence of an AU-rich element (ARE) within the 14 b fragment [81]. Another variation site at position +3187 may also influence HLA-G mRNA stability, with the presence of an Adenine associated with decreased mRNA stability* in vitro* and decreased HLA-G expression [78]. Interestingly, the SNP is found 4 bp upstream of an ARE. Furthermore, the nucleotide variation at the +3142 position was demonstrated to influence the binding of miR-148a, miR-148b, and miR-152 to the HLA-G messenger with an increased affinity in the presence of a Guanine instead of a Cytosine [83]. Despite a controversial data published by Manaster and colleagues [109], these results are consistent with the* in silico *analysis of microRNAs targeting the 3′ UTR [110]. Indeed, in addition to miR-148a, miR-148b, and miR-152, the binding ability of several miRNAs may potentially be influenced by the 3′UTR variations, emphasizing the role of the 14 bp indel, and SNPs at the +3003, +3010, +3027, and +3035 positions. Interestingly, we recently showed that most of 3′UTR SNPs are associated with differential amounts of sHLA-G in the plasma from healthy Brazilian and French populations [108]. In agreement with the known or putative specific impact of each variants, UTR-1 haplotype (14 bp Del, +3003T, +3010G, +3027C, +3035C, +3142C, +3187G, and +3196C) was classified as a high producer of sHLA-G while UTR-5 (14 bp In, +3003T, +3010C, +3027C, +3035T, +3142G, +3187A, and +3196C) and UTR-7 (14 bp In, +3003T, +3010C, +3027A, +3035T, +3142G, +3187A, and +3196C) were classified as low producers of sHLA-G [108].

Therefore in addition to microenvironment factors, HLA-G gene polymorphism is likely a relevant parameter to consider in tumoral HLA-G expression.

## 4. Concluding Remarks

**Figure 2:**
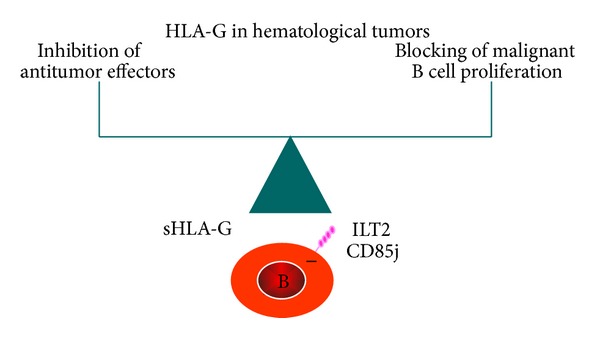
Dual role of HLA-G in hematological malignancies.

Based on the tolerogenic properties of HLA-G in the solid tumor context, blocking its expression and function may be considered as potential targets for antitumor therapy. One might either stop* HLA-G* transcription or interfere with its surface expression or secretion or block direct interaction between HLA-G expressed by tumor cells and inhibitory ILT receptors present on immune cells. Strategies aimed at blocking HLA-G expression by using RNA interference or HLA-G function by using specific antibodies may enhance the immune clearance of HLA-G^+^ tumor cells since all cell subsets involved in tumor rejection can express at least one receptor for HLA-G. In hematological malignancies, such as B cell malignancies, the role of HLA-G seems more complex and may depend on a balance between the HLA-G-driven inhibitory mechanism of antitumor responses and the antiproliferative effects on malignant B cells which will be under the control of ILT2 expression or of another as yet unknown receptor (Figure 2). Accordingly, considering ILT receptor expression and function in malignant hematopoietic cells will be of particular importance in understanding the mechanisms of tumor progression* in vivo*. In this regard, more clinical and basic studies are now required to establish a clear significance of HLA-G expression in hematological malignancies. Finally, precise determination of the nature of the HLA-G molecular structures/superstructures that are pathologically relevant* in vivo *as well asthe identification of individuals genetically prone to differentially express HLA-G in a specific environment will contribute to develop optimal HLA-G-based clinical strategies for diagnostic and therapy in cancer.
